# Ketogenic diet benefits body composition and well-being but not performance in a pilot case study of New Zealand endurance athletes

**DOI:** 10.1186/s12970-017-0180-0

**Published:** 2017-07-12

**Authors:** Caryn Zinn, Matthew Wood, Mikki Williden, Simon Chatterton, Ed Maunder

**Affiliations:** Auckland University of Technology (AUT). Sports Performance Research Institute New Zealand (SPRINZ). AUT Millennium, 17 Antares Place, Mairangi Bay, Auckland, New Zealand

**Keywords:** Low-carbohydrate, high-fat, LCHF, Endurance athletes, Well-being, Performance

## Abstract

**Background:**

Low-carbohydrate, high-fat and ketogenic diets are increasingly adopted by athletes for body composition and sports performance enhancements. However, as yet, there is no consensus on their efficacy in improving performance. There is also no comprehensive literature on athletes’ experiences while undertaking this diet. The purpose of this pilot work was two-fold: i. to examine the effects of a non-calorie controlled ketogenic diet on body composition and performance outcomes of endurance athletes, and ii. to evaluate the athletes’ experiences of the ketogenic diet during the 10-week intervention.

**Methods:**

Using a case study design, five New Zealand endurance athletes (4 females, 1 male) underwent a 10-week ketogenic dietary intervention. Body composition (sum of 8 skinfolds), performance indicators (time to exhaustion, VO_2_ max, peak power and ventilatory threshold), and gas exchange thresholds were measured at baseline and at 10 weeks. Mean change scores were calculated, and analysed using t-tests; Cohen’s effect sizes and 90% confidence limits were applied to quantify change. Individual interviews conducted at 5 weeks and a focus group at 10 weeks assessed athletes’ ketogenic diet experiences. Data was transcribed and analysed using thematic analysis.

**Results:**

All athletes increased their ability to utilise fat as a fuel source, including at higher exercise intensities. Mean body weight was reduced by 4 kg ± SD 3.1 (*p* = 0.046; effect size (ES):0.62), and sum of 8 skinfolds by 25.9 mm ± SD 6.9; ES: 1.27; *p* = 0.001). Mean time to exhaustion dropped by ~2 min (±SD 0.7; *p* = 0.004; ES: 0.53). Other performance outcomes showed mean reductions, with some increases or unchanged results in two individuals (VO2 Max: −1.69 ml.kg.min ± SD 3.4 (*p* = 0.63); peak power: -18 W ± SD 16.4 (*p* = 0.07), and VT2: -6 W ± SD 44.5 (*p* = 0.77). Athletes reported experiencing reduced energy levels initially, followed by a return of high levels thereafter, especially during exercise, but an inability to easily undertake high intense bouts. Each athlete reported experiencing enhanced well-being, included improved recovery, improvements in skin conditions and reduced inflammation.

**Conclusions:**

Despite performance decrements and some negative experiences, athletes were keen to pursue a modified low-carbohydrate, high-fat eating style moving forward due to the unexpected health benefits they experienced.

**Trial registration:**

ACTRN: ACTRN12617000613303. Registered 28 April 2017, retrospectively registered.

## Background

Low-carbohydrate, high-fat (LCHF) diets, including their extreme version i.e., ketogenic diets have recently become popular dietary regimes for athletes for several reasons. However, there is no consensus regarding the efficacy of ketogenic diets on sports performance. The overarching mainstream nutrition philosophy for endurance athletes, is one that emphasises a carbohydrate-dominant, low fat paradigm. Under these dietary conditions, athletes utilise carbohydrate as their predominant fuel source to fuel high volumes of aerobic exercise [1]. The appeal of LCHF eating for endurance athletes is likely due to the shift in fuel utilisation, from a carbohydrate-centric model to one that utilises fat predominantly, of which stores are unlimited compared to carbohydrate (i.e., muscle glycogen). This metabolic shift, seen after a period of dietary alteration is often referred to as being ‘fat-adapted’, which has been well-documented in studies since the 1980s [2, 3].

Despite the physiological advantage of utilising fat as a fuel source during sub-maximal exercise, to date there is no conclusive evidence to suggest that this results in subsequent performance enhancement [4]. Some individual responses to a ketogenic diet have shown dramatic benefits in both fat metabolism and performance, and are worth further investigation [5, 6]. However, studies have also shown a reduction in maximal aerobic performance [7] with some evidence indicating a negative effect on exercise intensity >70% of VO_2_ max [8, 9]. The majority of these studies have been criticised for not being long enough to allow for the full adaptive mechanisms to occur, which appear to require at least 21 days.

Despite the lack of peer-reviewed evidence for performance enhancement, athletes continue to be intrigued with the LCHF dietary paradigm. It is likely that this is the case for two reasons that go beyond the desire to obtain the extra performance edge: 1. a reduction in body fat that is frequently cited when athletes undertake LCHF or ketogenic diets [10], and 2. the anecdotal benefits that are cited by athletes eating this way. There is very little athlete-specific literature, particularly of a qualitative nature, addressing non-performance outcomes of LCHF or ketogenic eating in an athlete cohort. In a translational case study design, this pilot study set out to investigate the effect of a 10-week ketogenic diet on body composition and performance outcomes in five New Zealand endurance athletes, as well as to evaluate, qualitatively, the athletes’ experiences of undertaking the ketogenic diet during their training season.

## Methods

### Study design

This was a pilot, 10-week intervention undertaken with five case studies. A control group was not included. The study took place at AUT, Human Potential Centre; ethics was approved by the AUT Ethics Committee (application 15/415).

### Participants

Five recreational endurance athletes (four females, one male) consented to participate in this study. All participants were known to the primary investigator and were all highly motivated athletes regularly involved in high-level competitive endurance sport for at least five years. All participants were non-smokers, healthy and injury-free as per a health screening questionnaire and not consuming a low carbohydrate diet (defined as carbohydrates less than 45% of total energy intake). See Table 1 for participant demographics. The athletes were curious about the efficacy of the ketogenic diet on their body composition and sports performance. None of them had any previous experience with, or pre-conceptions about low carbohydrate or ketogenic diets prior to this trial.

**Table 1:** Participant demographics

Participant	Sex	Main sport	Training volume (hours/week)	Age (yr)	Weight (kg)	Height (cm)	∑8 Skinfolds (mm)
1	Female	Cycling	10–12	50	63.4	164.0	74.0
2	Male	Running	8–10	51	74.8	178.0	91.5
3	Female	Running	8–10	49	66.3	181.0	91.5
4	Female	Cycling	10–12	51	61.0	170.0	72.5
5	Female	Cycling	6–8	55	60.8	158.0	118.3

### Study protocol

Prior to the start of the intervention, participants underwent a series of tests and a full consultation for dietary instruction and planning as follows:

- *Performance test.* Participants reported to the lab in the morning in a fasted state and underwent a performance test on three occasions: i. one week prior to the intervention (familiarisation), ii. immediately prior to the intervention (baseline), and iii. Immediately post the intervention (post). To determine ${\mathrm{V}}^{.}{\mathrm{O}}_{2\mathrm{peak}}$ and gas exchange thresholds (GET) an incremental cycle test was performed using an electromagnetically controlled cycle ergometer (Ergoselect 100, Ergoline, Bitz, Germany) in a temperature-controlled laboratory (21 °C, 65% rH). The test commenced at 30 W and increased by 30 W every 3 min until volitional exhaustion. Participants were instructed to maintain a cadence of 80 revolutions.min^−1^. Oxygen uptake (${\mathrm{V}}^{.}{\mathrm{O}}_2$) was measured continuously using a breath-by-breath metabolic system (Metamax 3b, Cortex, Leipzig, Germany), and heart rate was continuously measured using a short-range telemetry device (Suunto M2, Suunto, Vantaa, Finland). The ${\mathrm{V}}^{.}{\mathrm{O}}_{2\mathrm{peak}}$ was defined as the highest 30s ${\mathrm{V}}^{.}{\mathrm{O}}_2$ value and the GET was identified independently by two experienced investigators using the V-slope method [11].

- *Dietary instruction.* An initial consultation was conducted with each participant by the primary researcher/Registered Dietitian directly after the performance test. Participants were provided with a daily macronutrient prescription of <50 g total carbohydrate, 1.5 g.kg protein and ad libitum fat. A detailed explanation of the practical application of the ketogenic dietary principles along with appropriate tracking and monitoring procedures was provided. Participants were provided with a sample diet and were advised to add variations as long as they adhered to the carbohydrate and protein macronutrient thresholds. They were instructed to track their diet using a dietary analysis programme Easy Diet Diary® (Xyris Software (Australia) Pty Ltd). Diets were monitored weekly by the primary researcher, and participants were contacted and offered additional support if they deviated. The primary researcher maintained regular contact with all participants throughout the 10-week study duration.

- *Exercise instruction.* The participants were all seasoned athletes, and participated on a regular basis in endurance events, i.e., mountain biking, road biking, running and multisport events (which included running, cycling and kayaking), both recreationally and competitively. Hence, their training protocols did not vary much from month-to-month. They were instructed to continue with their existing training volumes for the duration of the intervention.

- *Ketone blood testing.* Each participant was shown how to measure blood ketone levels via finger prick, and provided with a FreeStyle Optium ketone meter and ketone strips. They were instructed to measure their blood ketone levels daily between 2 pm and 4 pm. Nutritional ketosis was defined as a blood ketone (beta-hydroxybutyrate) level > 0.5 mmol/l.

- *Anthropometric testing.* Body weight and skinfolds were measured by an ISAK level one accredited anthropometrist, prior to and at the conclusion of the intervention, at the same time of the day. A sum of (∑) 8-site skinfold ISAK protocol was applied: triceps, biceps, subscapular, iliac crest, supraspinale, abdominal, front thigh and medial calf [12].

- *Interviews and focus group.* A 20–30 min individual interview was conducted on the phone during week 5 week and a 60-min focus group was conducted in person once the intervention had concluded. The importance of this qualitative work was to assess both the individual and the groups’ overall experiences of being on this diet for 10 weeks. The group session provided a chance for athletes to compare experiences and translate findings to future practice.

### Data analysis

Due to the explorative nature of this study, and our small sample size, quantitative data is presented as individual responses. Data was analysed using mean change scores, with Cohen’s effect sizes and associated confidence limits applied to quantify magnitude of change. We also elected to apply a probability statistic using a student’s t-test to determine the statistical meaning of the change. All statistics were generated and applied using Microsoft Excel 2016. We acknowledge that applying statistical models hold limited meaning in this context and it is not our intention to make any inferences about these outcomes to athlete populations. As such, outcomes with a significance level of *p* < 0.05 should be considered a trend only; and *p* < 0.01 significant only in the sense that further work is required to substantiate these findings. Interviews and the focus group were recorded and transcribed, after which data was analysed using thematic analysis. The data for the interviews and focus groups were combined and is presented as key themes with supporting transcripts.

## Results

### Diet and ketosis

The diets were geared to induce nutritional ketosis, which all the athletes achieved by the end of week 2. All athletes adhered to the macronutrient thresholds provided for the 10 weeks, apart from on two occasions during the first two weeks, once, where protein intake exceeded the recommended threshold over several days in one athlete and alcohol intake was excessive in another athlete, thereby preventing ketosis from being achieved. Table 2 presents an example of a day’s food intake for each participant along with the average energy and macronutrient breakdown for that day. The sample day was selected at random during week 5. The athletes varied little from the sample diets provided to them throughout the 10 weeks, with adherence to the diets verified by blood ketones always staying above 0.5 mmol/l from week 2 onwards. Blood ketones ranged from 0.5–4.2 mmol/l; females ranged 0.5–1.9 mmol/l, and never exceeded 1.9 mmol/l. The male athlete ranged 1.0–3.5 mmol/l, went below 1.0 mmol/l on two occasions (0.8 and 0.6 mmol/l) and measured 4.2 mmol/l on one occasion.

**Table 2:** Participants’ dietary data and macronutrient composition

Participant 1 (male)	Breakfast ½ cup granola^a^, 150 ml coconut cream, 100 g mixed frozen berries, 30 ml coconut oil	Lunch 125 g smoked salmon, 2 egg muffins^b^, 100 g avocado	Dinner 120 g fish cooked in 1 T^c^ olive oil, 80 g broccoli, ½ cup almonds, 50 g feta cheese	Other 2 X Coffee (60 ml cream) Water
	Energy 2450Cal; Net carbohydrate 24 g; Protein 103 g (1.4 g/kg); Fat 215 g			
Participant 2 (female)	Breakfast 3 rashers bacon, 2 eggs scrambled in 1 T coconut cream	Lunch Salad: spinach leaves, ¼ avocado, 30 g Gruyere cheese, 60 g salmon, spring onion, 1 T avocado oil, ½ cup sliced almonds	Dinner 100 g rump steak, salad (spinach, spring onion, pepper, carrot, desiccated coconut, 1 T pumpkin seeds), 2 tsp.^d^ pesto	Other 40 g blue cheese; 180 ml red wine; herbal tea; water
	Energy 1710Cal; Net carbohydrate 9 g; Protein 94 g (1.5 g/kg); Fat 128 g			
Participant 3 (female)	Breakfast ½ cup granola, 125 ml coconut cream, 50 g mixed frozen berries	Lunch 1 egg muffin, 100 g avocado, dessert spoon peanut butter, square chocolate	Dinner 100 g pork straps, 70 g spinach, 1 cup cauliflower rice, 1 T butter square 85% dark chocolate 180 ml red wine	Other Coffee, 100 ml soya milk; green tea; 1 strawberry; 20 g ham; 30 g almonds; water
	Energy 1768Cal; Net carbohydrate 33 g; Protein 76 g (1.1 g/kg); Fat 131 g			
Participant 4 (female)	Breakfast ½ cup granola^a^, 150 ml Greek yoghurt, 50 g mixed frozen berries	Lunch 95 g tin tuna, avocado, cheese, 1 T olive oil	Dinner 100 g chicken with skin, 1 tsp. soy sauce, 1 cup cauliflower, 25 g leek, 50 g asparagus, 1 T butter	Other 30 g salami; ½ cup almonds; coffee, 150 ml milk; 100 ml coconut cream, water
	Energy 1919Cal; Net carbohydrate 31 g; Protein 96 g (1.5 g/kg); Fat 154 g			
Participant 5 (female)	Breakfast 2-egg omelette (spinach, mushroom) cooked in 2 tsp. butter	Lunch Salad: 1 cup mesclun leaves, ½ avocado. 30 g feta cheese, 90 g chicken, 6 baby tomatoes, 50 g cucumber, 30 g celery, 2 T olive oil	Dinner 120 g fish, stir-fry vegetables (1 medium zuchini, 50 g broccoli, 5 mushrooms, ½ brown onion), cooked in 1 T olive oil	Snacks 100 ml coconut cream; 30 g almonds; water
	Energy 1406Cal; Net carbohydrate 19 g; Protein 93 g (1.5 g/kg); Fat 103 g			

^a^granola was made in a batch and consisted of a range of nuts, seeds, coconut threads and coconut oil

^b^egg muffins

^c^T = tablespoon

^d^tsp. = teaspoon

### Substrate oxidation

**Fig. 1:**
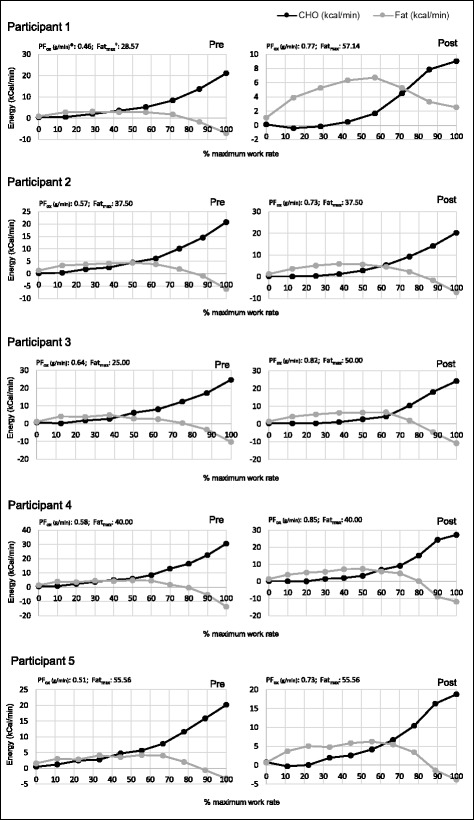
Pre, and post intervention metabolic efficiency curves for each participant. * PF_ox_: Peak Fat Oxidation (g/min). † Fat_max_: Maximum fat oxidation at % WR_max_

Figure 1 presents the pre and post intervention fuel utilisation (also termed metabolic efficiency) curves, along with associated peak fat oxidation (g/min) and fat max (%Wmax) for each participant.

Each curve displays the cross-over point i.e., the point at which peak fat oxidation is reached, and the point at which carbohydrate takes over as the predominant fuel source. Mean peak absolute fat oxidation and standard deviation (SD) increased by 41.3% (0.6 ± 0.1 to 0.8 ± 0.1 g.min^−1^, *p* = 0.001). All of the athletes increased their peak fat oxidation. The exercise intensity relative to ${\mathrm{V}}^{.}{\mathrm{O}}_{2 \max }$ at which peak absolute fat oxidation occurred (Fat_max_) increased by 31.2% from pre- to post-intervention (48.2 ± 8.7 to 63.2 ± 5.7%${\mathrm{V}}^{.}{\mathrm{O}}_{2 \max }$_,_ *p* = 0.06). Similarly, Fat_max_ relative to WR_max_ increased by 21.5% from pre- to post-intervention (39.5 ± 11.9 to 48.0 ± 8.9%WR_max_, *p* = 0.18). Two out of the five athletes showed an increase in Fat_max_ relative to WR _max_, and the remaining three showed no change.

### Body composition/performance

**Fig. 2:**
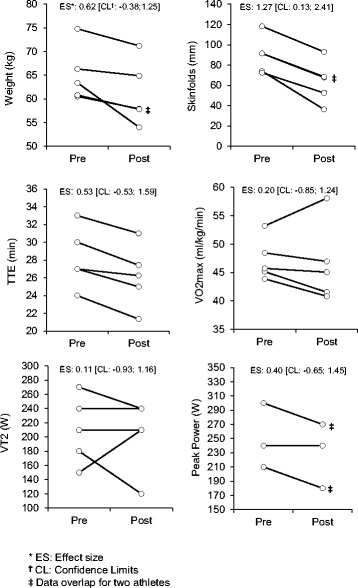
Individual responses and effect sizes of anthropometry and performance variables

Figure 2 shows individual pre and post scores for anthropometric and performance data. All five participants had reductions in body weight and skinfolds. The mean loss in weight and SD was −4.0 ± 3.1 kg (*p* = 0.046), and in skinfolds was −25.9 ± 6.9 mm (*p* = 0.001). The skinfold outcome showed a large effect size (1.27) with associated confidence limits not crossing zero, together indicating clinical significance in the context of their limited application to this data.

All participants showed a decrease in time to exhaustion (TTE), the mean time and SD reduction was 2 ± 0.7 min (*p* = 0.004). More varied responses were noted in the change in VO2 Max (mean change −1.69 ± 3.4 ml.kg.min (*p* = 0.63)), peak power (mean change −18 ± 16.4 W (*p* = 0.07)) and ventilatory threshold, VT2 (mean change −6 ± 44.5 W (*p* = 0.77)), with some athletes improving (VT2 and VO2 max) or staying the same (VT2 and peak power).

### Athlete experiences

Table 3 presents the key positive and negative themes extracted from the focus group. Overall, there were more positive themes than negative; with an improved sense of general health and well-being coming through as a strong theme. The majority of the negative experiences related to tiredness, experienced in the first few weeks of the intervention, and a feeling of loss of power, experienced towards the last few weeks. Each athlete reported the following subjective parameters: enhanced well-being, improved recovery, improved skin conditions and reduced inflammation.

**Table 3:** Themes from interviews and focus group with supporting transcripts

Positive themes	Negative themes
*Physical recovery*	*Loss of power during exercise/training*
“The recovery each time was amazing. I would go for a 4–5 h run then be able to mountain bike the next day, just to be able to do it without sore muscles”. “The next day after a 4-h run I could have got up and done it again - my legs weren’t tired or sore”. “Certainly I’ve had the recovery benefits I felt fine to go out the next day and do stuff I didn’t feel the muscle soreness I would have expected”. “I don’t feel sore”. “I was surprised that I could run comfortably for that long so I think the recovery was huge I never felt this, it’s unheard of”	“It was embarrassing to the point where I just got to the point where you just don’t have any energy”. (weeks 1–5) “I got too tired and I got to the point where I might have had some big runs, 4 or 5 h runs and wake in the night before hand, worried about it. I’d think, how am I going to do that tomorrow… it’s going to be hard”. (weeks 1–5) “The lack of power, I just couldn’t get up the hill.” (weeks 6–10)
	*Negative symptoms (weeks 1–3)*
	“I hadn’t been for 4 days and I was starting to get bound up”. (constipation). “One of the downsides is that I had cramps, massive cramps”. “I may have seemed a bit irritable for a while. People close to me realised I was down and I’m pretty good at hiding that but people picked it up”.
*Enjoyment of this way of eating*	
“It’s given me the freedom to eat the things that I stopped eating for a long time because I thought it was a no-no”. “Not feeling hungry has been amazing”. (X2) “Not being bothered about food, you know food doesn’t bother me anymore, as before I had a meal and 3 h later I’d feel irritable if I didn’t eat”. “It’s been fantastic for me having food I couldn’t for a long time and I really enjoy it, the flavour, for me the reason why I want to carry on with this way of eating is because I actually enjoy it, I enjoy it far more than my previous diet”.	
	*Food regime boredom*
	“Bored with the food regime, actually I’m back on fruit …” “…but I am starting to get sick of the diet plan”. “You can’t even free load in your veggies.”
*Weight management*	
“Being able to shed that 5 kg was definitely a benefit”. “The benefit now of me being able to understanding how to manage my weight, that’s been a real effect, actually that’s been for all of us”.	
*Feel better overall (well-being)*	
“My skin hasn’t been this good since I can remember”. “My prostate, a million times better because I can sleep”. “The other thing I noticed is my tinnitus is a lot better, I can’t hear the ringing in my ears I used to have”. “Just general health - I just feel better, I need less sleep”. “I noticed that I didn’t fart for 2 months, no gas”. “Yes, no gas, that’s the same as me.” “I feel sharper mentally”. “Food used to dictate my moods, and now it doesn’t”. “Positive skin, I have a facial every 6 weeks, I didn’t tell the facial lady what I was doing and she said to me what’s happened to your skin? No extractions in the last 8 weeks, no blockages”.	

## Discussion

This study presents a real-life insight into the lives of five seasonal endurance athletes who by virtue of their own curiosity wished to experience the effects of a ketogenic diet on their sports performance. Overall, participants were able to increase the substrate utilisation of free fatty acids, reduce body fat and experience positive health benefits, but their maximal aerobic performance was compromised.

### Body composition

The reduced body fat can likely be explained by a resultant calorie deficit created by the diet, as participants reported enhanced feelings of satiety and a reduction in overall food intake. This outcome was unsurprising and comparable to findings in previous research on both strength and endurance athletes [13–15]. Initial weight reduction can be associated with a loss in body water through glycogen depletion, [16, 17], and this was also likely the case in this study; however fat loss was evident as per skinfold changes. A further theory relating to weight loss, which is as yet, rigorously tested, is an increased drive for fat breakdown rather than storage as circulating levels of insulin remain low during ketogenic diets [18]. Perhaps a combination of all three mechanisms can explain the weight loss. A limitation of the study was a lack of energy comparison prior to, and during the study, which would have provided some clarity about these mechanisms.

### Metabolic efficiency

All participants had a greater fatty acid oxidation at a higher given intensity at the end of the trial compared to baseline. This finding of enhanced fat utilisation aligns with those of several other groups that have incorporated ketogenic and non-ketogenic dietary protocols [5, 19, 20]. Furthermore, this substrate utilisation alteration can be attributed to the change in diet as training was kept relatively consistent throughout the intervention. Our participants also had a higher oxygen cost at sub-maximal workloads due to the higher use of fat as an energy substrate. However, this did not benefit exercise capacity.

### Performance

On the whole, maximal aerobic performance was reduced, another comparable outcome to similar research [21–23], the exception being one athlete in Phinney et al.’s 4-week cycling study [5], and Zajac et al.’s eight off-road cyclists [6], who all showed performance increases. The performance decrement in our study, and others, is likely due to changes in metabolic pathways that impair glycogen metabolism at higher exercise intensities [24–26]. Specifically, a down-regulation of the carbohydrate oxidative enzyme, pyruvate dehydrogenase (PDH), which via conversion of pyruvate to acetyl-coenzyme A, links the glycolytic pathway with the Krebs cycle [27]. PDH is said to be reduced rapidly through a reduction in circulating insulin and an increase in circulating levels of free fatty acids [28]. Evidence suggests that PDH is upregulated upon carbohydrate reintroduction [25]; however, there is little insight into its fate along with other mitochondrial enzymes in the context of low carbohydrate availability. Despite similarities in findings with other studies, some of these studies are limited by short-duration low carbohydrate diets [28–30]. Future research with chronically fat-adapted athletes is needed to investigate these micro-level mechanisms alongside performance outcomes. Recently, Volek et al.’s work [19] with chronically fat-adapted ultra-endurance athletes (>6 months) not only demonstrated a 2.3 times greater fat oxidation rate in the LCHF group compared with the mainstream dietary group, but also demonstrated no difference in resting and replete muscle glycogen stores between groups. Authors suggest a homeostatic muscle glycogen repletion mechanism arising from hepatic gluconeogenesis, which might serve to provide clues into why many athletes report optimal performance, anecdotally, when having eaten in an LCHF manner for extended periods of time. While this is a plausible speculation, a similar study by Webster et al., [20] showed no difference in gluconeogenic rates during exercise in fasting LCHF and mixed-diet athletes. In fact, glucose was produced endogenously to a greater extent in the mixed diet group, and was attributed to greater rates of hepatic glycogenolysis. Researchers concluded that gluconeogenesis during exercise may remain stable across a range of dietary regimes after an overnight fast, but that hepatic glycogenolysis is influenced by dietary carbohydrate. Further exploration of fuel contributions to gluconeogenesis and the effect of different feeding protocols on endogenous glucose producing mechanisms is warranted. It is important to note that both of these studies did not incorporate a performance measure, leaving the questions to this key issue unanswered [19, 20].

### Athlete experiences

This is one of the few studies to report specifically on endurance athletes’ experiences of undertaking a ketogenic diet. Athletes reported similar negative physiological experiences to those reported by athletes in comparable ketogenic diet studies [13, 14, 31]. However, they also reported experiencing benefits throughout the trial. One of these benefits was enhanced recovery; possibly, the rise in blood ketones had some influence, as beta-hydroxybutyrate has been associated with upregulating antioxidant gene expression and decreasing reactive oxygen species [32]. However, further research is required to substantiate this within athletic populations.

From a physical well-being perspective, the cases of improved skin, and the resolution of an ongoing prostate issue, were major points of discussion of benefits experienced. We speculate that it is the reduction of systemic inflammation as a result of a lower total sugar [33] and Omega 6 fatty acid intake, thereby rebalancing the Omega 6:3 fatty acid ratio in an anti-inflammatory direction [34] that gave rise to these outcomes. All participants were consuming high-Omega 6 industrial seed oils prior to the study (used as cooking fat and derived from processed foods). During the study these fats were replaced with coconut oil, butter and olive oil; i.e., fats containing minimal Omega 6 fatty acid content.

Being a translational study, we followed up participants informally 12 months after the study concluded. They were all still competing in endurance events, and while not eating a ketogenic diet, none of them had returned to their previous high carbohydrate, low fat style of eating. Collectively, they reported that once the study concluded they gradually increased their carbohydrate intake until the point at which they felt their performance at high intensities return. They were still restricting carbohydrate and eating more fat than mainstream guidelines recommend, and reported having discovered the optimal macronutrient ratio that satisfied a performance, body composition and a health goal.

This study had several limitations: Its design as a pilot case study, with no standardisation of training prevents any inference from being made to athletic populations. However, it is still relevant to both the researcher and the practitioner as it provides insights into what is considered important for athletes, particularly those in the 40+ age range. i.e., alongside improving performance, they are also more cognisant of their overall health and well-being. A lesson learned from undertaking this research, and a key consideration for researchers and practitioners, is to encourage a reduction in athlete training intensity and volume in the early weeks of embarking on a ketogenic diet. This will likely induce less early fatigue and other negative symptoms related to training, and allow for metabolic adaptations to occur in a lower stress milieu.

## Conclusion

Despite a decrease in performance, athletes reduced body fat and experienced unexpected well-being benefits. While performance outcomes are key to the field of sports science and medicine, what might be overlooked, is the integration of health and well-being, alongside performance. Further research, both conceptual and translational, should challenge this type of diet further to understand its potential uses to achieve what an endurance athlete and their support team should ultimately be striving for i.e., optimal body composition, health and performance.

## Abbreviations

ES: Effect size
GET: Gas exchange thresholds
LCHF: Low-carbohydrate, high-fat
PDH: Pyruvate dehydrogenase
TTE: Time to exhaustion
